# Sexual health and human rights: protecting rights to promote health

**DOI:** 10.1186/s12879-019-3860-3

**Published:** 2019-03-06

**Authors:** Joseph D. Tucker, Benjamin M. Meier, Cecilia Devoto, Eva Szunyogova, Stefan Baral

**Affiliations:** 1 Institute of Global Health and Infectious Diseases, University of North Carolina at Chapel Hill, 130 Mason Farm Road, Bioinformatics Building, Chapel Hill, NC USA; 2 Social Entrepreneurship to Spur Health (SESH), Guangzhou, China; 3 Faculty of Infectious and Tropical Diseases, London School of Hygiene and Tropical Medicine, London, UK; 4 Department of Public Policy, University of North Carolina at Chapel Hill, Chapel Hill, NC USA; 5 Department of Health Policy and Management, Gillings School of Global Public Health, University of North Carolina at Chapel Hill, Chapel Hill, NC USA; 6 O’Neill Institute for National and Global Health Law, Georgetown Law Center, Washington, DC USA; 7 BMC Infectious Diseases, BMC, London, UK; 8 BMC International Health and Human Rights, BMC, London, UK; 9 Departments of Epidemiology, International Health, and Health Policy and Management, Bloomberg Johns Hopkins School of Public Health, Baltimore, MD USA; 10 Center for Public Health and Human Rights, Johns Hopkins School of Public Health, Baltimore, MD USA

Where sexual health refers to “a state of physical, mental, and social well-being in relation to sexuality,” [1] human rights provide a path to realise the highest attainable standard of sexual health for all [2]. Jonathan Mann first described the fundamental relationship between sexual health and human rights in the early years of the global AIDS response. He traced the arc between rights abuses and HIV transmission and provided a path to see sexual health and human rights as inextricably linked [3]. The close interrelationships between sexual health and human rights led to the development of an evidence-based but rights-affirming approach to sexual health, abandoning coercive tools of public health, reducing stigma and discrimination toward key populations, and focusing on the social determinants of sexually transmitted infections (STI) [4]. Sexual health is thus seen as deeply embedded in the social and structural fabric of societies, with rights-based implications for epidemiology, intervention design, behavioral science, harm reduction, and laws and regulations governing health. Yet, many evidence-based STI interventions are unavailable because those at greatest risk hide their lives and sexual desires and practices from clinics and services. This BMC special collection focuses on the critical nexus of sexual health and human rights.

This topic was chosen because of three related trends – social determinants of sexual health, human rights implications of public health programming, and limitations of sexual health approaches that exclude rights. First, there is a broad and growing literature that seeks to understand how social determinants influence STI spread. For example, policing practices such as confiscating condoms among sex workers can profoundly shape the risk environment, increasing the risk of STI transmission [5]. On the other hand, respecting, protecting, and fulfilling the rights of key populations can decrease STI risk. An ecological analysis of gay civil unions in Europe found that passing gay civil union laws was associated with a 24% reduction in reported syphilis cases [6]. Second, human rights address basic needs and frame individual entitlements, conceptualizing universal frameworks to advance justice in public health [7]. Sexual rights are inherently important to human dignity and at the same time, instrumentally important to public health. Finally, there are clear limitations to public health programs that exclude rights. Many studies have found that programs ignoring human rights or related structural issues in sexual health ultimately miss their mark. Effective sexual health interventions require integration of biomedical, social and structural insights.

This special issue is a collaboration between *BMC Infectious Diseases* and *BMC International Health and Human Rights*. It includes 13 original research articles and one debate article drawing attention to human rights barriers in accessing sexual health services, as well as the need for better measures to protect human rights, minimize discrimination, and promote sexual health.

Gay men and other cisgender men who have sex with men (MSM), cisgender female sex workers (FSWs), transgender populations, and other key populations face substantial stigma, discrimination and gender-based violence (GBV), which are known to influence mental health and sexual risk practices. MSM have been shown to be at high risk of HIV infection. The study by Takano et al. observes a 3% prevalence of HIV infection among MSM in Tokyo [8].

Despite extensive research on the human rights abuses underlying sexual health, limited research has characterized the types and sources of stigma. Grosso et al. analyse factors related to stigma among MSM and FSWs in Sub-Saharan Africa [9]. These includes experiences such as being arrested, verbally harassed, blackmailed, or physically abused. Stigma is linked to healthcare workers, family, friends, and the police. Healthcare related stigma includes situations where patients are denied care, provided suboptimal care, or gossiped about by healthcare workers.

In another study, Lyons et al. show that sexual behavior stigma at a community level is associated with individual-level determinants of HIV and STI risks among MSM and transgender women in eSwatini [10]. These associations vary by level of outness about sexual practices. They suggest that achieving sufficient coverage of evidence-based stigma mitigation interventions will be critical for realizing the impact of HIV prevention and treatment interventions.

Social stigma is not the only barrier to effective treatment for sexually transmitted and blood borne infections (STBBIs). Other factors, such as policies and laws criminalizing same sex practices, play an important role. These are highlighted in research by Strömdahl et al., showing high levels of experienced stigma, discrimination and human rights violations among MSM in Abuja, Nigeria [11]. Given the increased incidence of HIV/STIs in the country, their findings reinforce the need for structural interventions to facilitate access to services. This need is further emphasised by Argento et al., who show multilevel factors that potentiate and mitigate STBBI risk for sex workers of all genders [12]. Their data suggest that multipronged structural and community-led approaches are paramount in addressing the STBBI burden, and are necessary to realizing the health and human rights of sex workers. In a second review, Stangl et al. present encouraging evidence of human rights interventions that enable a comprehensive HIV response [13]. The best available evidence suggests that scaling up human rights programs and rights-based interventions is a critical component to increase and sustain engagement in evidence-based HIV prevention and treatment services. Similar conclusions can be drawn from the study by Evens et al., focused on understanding the nature and consequences of GBV affecting key populations across Latin America and the Caribbean [14]. Their data suggest that coordinated interventions that address both HIV and GBV are essential in reducing the national burden of HIV while also promoting key populations’ human rights.

In HIV prevention research, key populations represent a wide range of different populations with varying risks of HIV or STIs, highlighting the need for differentiated approaches in response to specific needs [15]. Latent class analysis (LCA) has recently emerged as a popular approach to identify subgroups in a given population. However, the generalizability of emergent population structures across different settings has yet to be considered. Smith et al. compared LCA performed on two online samples of HIV negative Chinese MSM to detect latent class structures and assessed the extent to which sampling considerations impact the validity of LCA results [16]. By combining results from two simultaneous LCAs conducted on distinct samples of Chinese MSM, their analysis provide more robust insights than would have been possible from a single LCA. Furthermore, their results may not only serve as a template for future LCAs, but also provide guidance in ways to strengthen methodological approaches to mapping and characterizing HIV risk.

Globally, access to HIV services for cisgender MSM and FSWs remains suboptimal; however, little is known about the perceptions of health care workers in providing HIV services to MSM and FSWs. In the article by Matovu et al., the authors explore the concept of stigma among health care workers in Uganda and report that while most health care workers are comfortable providing HIV services to FSWs, there were strong homonegative tendencies towards MSM [17]. Some health providers reported that they “would feel very uncomfortable” evaluating MSM because they engage in “a culture imported into our country.” A majority of the health providers felt that they did not have adequate skills to effectively serve MSM and called for specific training to improve their clinical skills.

As a result of the issues raised by Matovu et al., MSM are less likely to use community sexual health facilities. Depression and stigma may impact on health care utilization among this group. In a cross-sectional study of 1301 MSM aged 18 years and older in Abidjan, Yamoussoukro, Gagnoa and Bouake, Cote d’Ivoire, Ulanja et al. analyse the association between depression and health care utilization, with utilization represented by HIV and STI testing and treatment [18]. They conclude that evidence-based mental and sexual health services need to be integrated with stigma mitigation interventions to reduce health disparities among MSM in Cote d’Ivoire.

In countries such as Brazil, stigma within health services is widely observed among FSWs, and this may be detrimental to health seeking attitudes and practices amongst this key population. In the study by Dourado et al., the authors highlight how sex work stigmatization within health services may be one of the main barriers to FSW STI/HIV responses [19]. Their research underlines the importance of combating stigmatization and discrimination affecting FSWs in health services in order to guarantee the appropriate uptake of preventive methods available in the public health system in Brazil.

Stigma is a complex construct and includes both enacted and internalized stigma. Internalized HIV stigma is an outcome of perceptions and anticipations of stigma that can compromise engagement in HIV prevention, care and treatment. Internalized stigma is common given heteronormative societies, but there remain few well-established programs to address this stigma. In the debate article by Pantelic et al., the authors aim to initiate a dialogue to remedy this gap in knowledge and highlight the urgent need for designing interventions that take into account ethical considerations, the evolution of stigma theory over the past five decades, intersectionality of multiple stigmas, and opportunity cost [20].

Nakku-Joloba et al. examine factors influencing male partners to seek treatment after syphilis notification by their pregnant partners [21]. Despite the fact that syphilis screening can be successfully integrated into antenatal clinics, male partners seldom return to the clinic for treatment after notification by their infected pregnant partners. Reasons for this lack of follow-up include fear of injection pain, perceptions of syphilis as a genetic disease and a woman’s problem, busy work schedules, poor access to high-quality STI services, shared facilities with women in clinics, and HIV-related stigma. The authors conclude that improved public messaging about syphilis (to minimise intersectional stigmas), as well as infrastructural adjustments for improved access to STI care, legal and policy frameworks that support STI notification and treatment in resource-constrained settings are needed for effective STI control.

Other key populations that face difficulties accessing health services due to stigma are migrants from sub-Saharan Africa (misSA). As reported in the study by Müllerschön et al., misSA, specifically those without health insurance, face many barriers to accessing health care and HIV testing services due to their residence status and cultural, socioeconomic, legal and linguistic barriers [22]. The study concludes that having no health insurance or medical treatment voucher decreases the odds of contact with the healthcare system more than other socio-demographic characteristics. Furthermore, misSA without health insurance have lower odds of ever having done an HIV test compared to participants with health insurance.

This special issue highlights several research gaps related to implementation science, low- and middle-income countries, and participatory research. From the perspective of implementation science, empirical research is necessary to examine the effects of specific rights-based policies on specific clinical outcomes. Using the Mann framework [3], it is crucial to invest in study of the effects of human rights violations on health, public health policies on human rights violations and human rights protections on public health promotion. With limited investment in human rights-related research, the gap in knowledge across low and middle-income countries limits policy and program improvements for those most marginalized. In these contexts, achieving an AIDS-free generation in just a decade seems impossible. Advancing rights-based interventions and supporting participatory research among key populations remains central to achieving the vision laid out by Jonathan Mann at the outset of the health and human rights movement. Now is the time to protect human rights in order to promote sexual health.

## Abbreviations

FSW: Female sex worker
GBV: Gender-based violence
LCA: Latent class analysis
misSA: migrants from sub-Saharan Africa
MSM: Men who have sex with men
STBBIs: Sexually Transmitted and blood borne infections
STI: Sexually transmitted infections
